# Dual Mechanisms of Coniferyl Alcohol in Phenylpropanoid Pathway Regulation

**DOI:** 10.3389/fpls.2022.896540

**Published:** 2022-05-06

**Authors:** Mengling Guan, Changxuan Li, Xiaotong Shan, Fang Chen, Shufang Wang, Richard A. Dixon, Qiao Zhao

**Affiliations:** ^1^Shenzhen Key Laboratory of Synthetic Genomics, Guangdong Provincial Key Laboratory of Synthetic Genomics, Shenzhen Institute of Synthetic Biology, Shenzhen Institute of Advanced Technology, Chinese Academy of Sciences, Shenzhen, China; ^2^Center for Plant Biology, School of Life Sciences, Tsinghua University, Beijing, China; ^3^Department of Biological Sciences, University of North Texas, Denton, TX, United States; ^4^BioDiscovery Institute, University of North Texas, Denton, TX, United States; ^5^Beijing Advanced Innovation Center for Tree Breeding by Molecular Design, Beijing Forestry University, Beijing, China

**Keywords:** phenylpropanoid pathway, coniferyl alcohol, *KFB01/20/39/50* expression, PAL stability, signaling molecule

## Abstract

Lignin is a complex phenolic polymer that imparts cell wall strength, facilitates water transport and functions as a physical barrier to pathogens in all vascular plants. Lignin biosynthesis is a carbon-consuming, non-reversible process, which requires tight regulation. Here, we report that a major monomer unit of the lignin polymer can function as a signal molecule to trigger proteolysis of the enzyme L-phenylalanine ammonia-lyase, the entry point into the lignin biosynthetic pathway, and feedback regulate the expression levels of lignin biosynthetic genes. These findings highlight the highly complex regulation of lignin biosynthesis and shed light on the biological importance of monolignols as signaling molecules.

## Introduction

The phenolic polymer lignin, along with the polysaccharides cellulose and hemicellulose, are the major components of the secondary cell wall in vascular plants. Lignin is essential for providing strength and hydrophobicity to secondary cell walls. It is synthesized from deamination of the amino acid L-phenylalanine (Phe), which also serves as precursor of numerous specialized metabolites (Bonawitz and Chapple, 2010), by L-phenylalanine ammonia-lyase (PAL). A series of aromatic hydroxylations, *O*-methylations and side-chain reductions then lead to the formation of the three major lignin monomers (monolignols) *p*-coumaryl alcohol, coniferyl alcohol (CA) and sinapyl alcohol (Bonawitz and Chapple, 2010). After transport across the plasma membrane, most likely by a passive diffusion mechanism (Perkins et al., 2022; Zhuo et al., 2022), monolignols are oxidatively polymerized by laccases and peroxidases in the apoplastic space (Lee et al., 2013; Zhao et al., 2013).

The biosynthesis of lignin consumes approximately 30% of photosynthetically fixed carbon and is not reversible (Boerjan et al., 2003). Therefore, carbon resources that are channeled through Phe toward the biosynthesis of lignin must be tightly controlled to ensure that the Phe pool remains sufficient for protein synthesis. The regulation of lignin biosynthesis occurs at transcriptional, translational and post-translational levels (Taylor-Teeples et al., 2015; Zhao, 2016; Chezem et al., 2017; Geng et al., 2020). It has been well-documented that most lignin biosynthetic genes contain the so-called AC elements, including AC-I (ACCTACC), AC-II (ACCAACC), and AC-III (ACCTAAC) in the promoters that are recognized by MYB transcription factors (Raes et al., 2003). In Arabidopsis, MYB58 and MYB63 are the major transcription activators essential for lignin biosynthesis (Zhou et al., 2009). Recently, MYB20, MYB42, MYB43, and MYB85 have been identified as novel transcriptional activators that not only directly regulate the expression of the lignin biosynthetic genes but also the genes involved in the production of phenylalanine, which serves as precursor of lignin biosynthesis (Geng et al., 2020). Members of subfamily 4 of the R2R3-MYB family have been reported to function as transcriptional repressors of lignin biosynthesis. AmMYB308 and AmMYB330 in *Antirrhinum*, MYB4 in Arabidopsis, EgMYB1 in *Eucalyptus gunnii* and ZmMYB31 and ZmMYB42 in maize have all been shown to repress lignin biosynthetic genes by directly binding to AC *cis*-elements (Tamagnone et al., 1998; Jin et al., 2000; Fornalé et al., 2010).

Plant natural product biosynthesis is known to feature metabolite-mediated regulation. For instance, most amino acid biosynthetic pathways are subjected to tight feedback regulation (Galili et al., 2016). In the aromatic amino acids (AAA) biosynthesis pathway, the enzyme mediating the entry reaction is regulated by the final products (Maeda and Dudareva, 2012). Recently it has been reported that disruption of *DHS1* and *DHS3* caused hypersensitivity to tyrosine and tryptophan, respectively, while the *dhs2* mutant was resistant to tyrosine-mediated growth inhibition (Yokoyama et al., 2021). This suggests that the AAA biosynthesis pathway is regulated by complex metabolite-mediated mechanisms. However, the underlying molecular nature of the regulation is not clear.

Although it is important for plants to tightly control lignin biosynthesis to avoid overconsumption of Phe, how plants monitor lignin biosynthesis remains elusive. It is intuitive that the regulation of lignin biosynthesis might involve sensing of lignin-specific precursors. It was recently shown that CA biosynthesis can be maintained in the absence of guaiacyl or syringyl lignin biosynthesis (Zhuo et al., 2022), suggesting that CA may serve roles beyond simply being a lignin building block. In this study, we explored the function of CA in the regulation of lignin biosynthesis. We showed that CA treatment rapidly induces the expression of the genes encoding Kelch repeat F-box proteins, which mediate PAL stability. In addition, the expression of the lignin biosynthetic genes was subjected to feedback regulation by CA. Together, these data suggested that the major monolignol CA can regulate lignin biosynthesis in multiple ways.

## Materials and Methods

### Plant Materials and Growth Conditions

All Arabidopsis (*Arabidopsis thaliana*) lines used were in the Col-0 ecotype background. The *kfb39* (SALKseq_076098.2), *pal2* (Wiscseq_DsLoxHs159_03B), *pal3* (SALKseq_030894), and *pal4* (GABI_149A05) *Arabidopsis thaliana* mutants were obtained from the Arabidopsis Biological Resource Center (ABRC). The *kfb01/20/39/50* quadruple mutant was isolated from the progenies of a cross between the previously described *kfb01/20/50* (Zhang et al., 2015) and *kfb39*. The *kfb01/20* and *kfb39/50* were isolated from the progenies of a cross between Col-0 and *kfb01/20/39/50*. All materials used in this study including Col-0 and mutants were grown on half-strength (1/2) Murashige and Skoog (MS) medium containing 1.5% sucrose and 0.35% Phytagel (pH 5.8) at 22°C under long-day conditions with a 16-h light/8-h dark cycle.

To investigate the effects of monolignols and vanillyl alcohol (VA) on *A. thaliana*, 2-week-old seedlings were treated in 1/2 MS liquid medium containing 0.1 mM monolignol or 0.1 mM VA (with an equal volume of solvent as the control) for different time periods before qRT-PCR analysis and PAL stability assay.

### Plasmid Construction

The CaMV 35S promoter and the coding sequence of *YFP* were amplified and cloned into *pCAMBIA1300* to generate *35Spro:YFP*, and then the full-length coding sequences of *KFB01*, *KFB20*, *KFB39*, and *KFB50* without the stop codon were separately amplified and cloned into *35Spro:YFP* to obtain *35Spro:KFB01-YFP*, *35Spro:KFB20-YFP*, *35Spro:KFB39-YFP*, and *35Spro:KFB50-YFP* constructs. To obtain the truncated KFBs-YFP, the DNA fragments encoding KFB01_Δ1–35_, KFB20_Δ1–8_, KFB39_Δ1–50_, and KFB50_Δ1–31_ were separately amplified and cloned into *35Spro:YFP*.

To obtain the *UBQ10pro:PAL1-HA*, *UBQ10pro:PAL2-HA*, *UBQ10pro:PAL3-HA* and *UBQ10pro:PAL4-HA*, the *UBQ10* promoter was cloned into *pEarlyGate 301* to generate the *UBQ10pro:HA* construct, and then the coding sequences of *PAL1*, *PAL2*, *PAL3*, and *PAL4* without the stop codon were separately amplified and cloned into *UBQ10pro:HA*. For Co-IP assay, the KFBs-YFP and PALs-HA constructs were separately transformed into *Agrobacterium tumefaciens* strain GV3101, which was used to transiently express proteins in *Nicotiana benthamiana* leaves by the injection method.

### Monolignol Metabolite Quantification

To determine the monolignol metabolites, 3-week-old Arabidopsis seedlings were separated into leaf and root samples, and inflorescence stems were collected from plants that had just started to flower. Tissues were harvested and ground in liquid nitrogen. The samples (∼120 mg) were extracted with 100% methanol (500 mL) in a cold room for 1 h, vortexed briefly and extracted at room temperature for another hour. The liquid phase was separated by centrifugation at 13,000 *g* for 10 min and passed through a 0.45 mm minifilter before LCMS analysis. For each sample, materials from about 15 plants were pooled. Two biological replicates were processed for each tissue. The strategy for monolignol quantification was as previously described (Cocuron et al., 2019). Specifically, phenolic compounds were detected and quantified using an Agilent 1290 Infinity II liquid chromatography system coupled to a hybrid Triple Quadrupole 6500 + triple quadrupole from AB Sciex. The extracts were kept at 4°C in an auto-sampler. The metabolites were resolved at 30°C using a reverse phase C18 Symmetry column (4.6 × 75 mm; 3.5 μm) associated with a Symmetry C18 pre-column (3.9 × 20 mm; 5 μm) from Waters. The liquid chromatography gradient was made of 0.1% (v/v) acetic acid in acetonitrile (A) and 0.1% (v/v) acetic acid in water (B). The total LC-MS/MS run was 15 min with a flow rate of 800 μL/min. The following gradient was applied to separate the phenolic compounds: B = 0–1 min 85%, 1–7 min 42%, 7–9 min 20%, 9–9.1 min 15%, 9.1–12 min 15%, 12–12.1 min 85%, 12.1–15 min 85%. The injection needle was rinsed with 50% aqueous methanol. Five μL of extracts or external standard mixture was injected onto the column.

The detection of monolignols was accomplished using an AB Sciex hybrid Triple Quadrupole/Ion trap mass spectrometer QTRAP 6500+. Electrospray ionization with polarity switch was utilized to acquire mass spectra of the phenolic compounds. In this study, negative ionization (–4500 V) was applied to *p*-coumaraldehyde, *p*-coumaryl alcohol, sinapyl alcohol, whereas positive ionization (5000 V) was used for coniferaldehyde, CA, and sinapaldehyde. The settling time between each polarity was 15 ms. Metabolites were simultaneously detected as precursor ion/product ion pair using multiple reaction monitoring (MRM): coniferaldehyde (m/z 179 > 147); CA (m/z 163 > 131); *p*-coumaraldehyde (m/z 147 > 119); *p*-coumaryl alcohol (m/z 149 > 131); sinapaldehyde (m/z 209 > 177); sinapyl alcohol (m/z 209 > 194). The source parameters such as curtain gas (40 psi), temperature (550°C), nebulizer gas (50 psi), heating gas (60 psi), and collision activated dissociation (Medium) were kept constant during MRM. The dwell time in the mass spectrometer was set to 20 ms. Analyst 1.7 software from AB Sciex was used to record and process the LC-MS/MS data. Monolignols were identified and quantified using a mixture of known external standards run at the same time as the biological extracts.

### RNA Analysis

For the RNA-seq analysis, 2-week-old Arabidopsis seedlings of ecotype Col-0 were separately treated with 0.1 mM CA (or EtOH control) for 15, 30 min, 1 and 4 h, and then the seedlings from each treatment were divided into three biological replicates. The total RNA of each sample was extracted using the RNeasy MinElute Cleanup Kit (74204, Qiagen, Germany) before sending to BGI Genomics (The Beijing Genomics Institute) for RNA-seq analysis.

For qRT-PCR analysis, total RNA was isolated using TRIzol™ Reagent (15596018, Invitrogen, United States) and treated with DNase I (AMPD1-1KT, Sigma-Aldrich, United States). Complementary DNA (cDNA) was obtained using SuperScript™ III Reverse Transcriptase (18080093, Invitrogen, United States). The qRT-PCR reaction was set with TB Green™ Premix Ex Taq™ II (RR820L, Takara, China). *GAPDH* was used as the control gene for normalization. The primers used for qRT-PCR analysis are listed in Supplementary Table 1.

For RT-PCR analysis, equal quantities of the total RNA extracted from 2-week-old seedlings of Col-0 and *kfb01/20/39/50* were reverse-transcribed. The RT-PCR was performed with Taq DNA Polymerase (AP111-02, Transgen, China), and *TUA4* was used as the control gene. Primers are listed in Supplementary Table 1.

### Co-immunoprecipitation Assays

Tobacco leaves transiently expressing target proteins were harvested 48 h after infiltration, and then the total proteins were extracted with 50 mM Tris-HCl buffer, pH 7.5, containing 150 mM NaCl, 10% glycerol, 5 mM MgCl2, 0.1% NP-40, 1 × complete protease inhibitor cocktail, 5 mM DTT and 60 μM MG132. GFP-Trap agarose beads incubated with the crude protein extracts were rotated for 1 h at 4°C. The protein-bound beads were washed for five times, and then boiled for 5 min after mixing with 80 μL protein extraction buffer (50 mM Tris-HCl buffer, pH 7.5) and 20 μL 5 × SDS-PAGE sample loading buffer. Protein samples were separated by SDS-PAGE gel and the immunoblot analyses were performed using monoclonal anti-GFP antibody (11814460001, Roche, China) or monoclonal anti-HA antibody (12013819001, Roche, China).

### Endogenous L-Phenylalanine Ammonia-Lyase Stability Analysis

The seeds of Col-0 and mutants were sown on 1/2 MS solid medium. After 7 days, the seedlings were transferred sparsely to other culture plates and grown for an additional 1 week. Subsequently, the seedlings were submerged in 1/2 MS liquid medium containing DMSO, 0.1 mM CA or 0.1 mM vanillyl alcohol (VA) and cultivated for different time periods. Seedlings after treatment were harvested and ground into powder in liquid nitrogen, then an equal volume of plant powder from each treatment was taken and separately mixed with 2 × SDS-PAGE sample loading buffer and boiled for 5 min. After centrifugation, protein supernatants were harvested and then separated by 10% SDS-PAGE gel. After electrophoresis, the proteins on the SDS-PAGE gel were electrophoretically transferred onto a polyvinylidene difluoride (PVDF) membrane (IPVH00010, Millipore). The Rubisco large subunit (RbcL) band was used as internal reference and the PVDF membrane was stained with Ponceau S solution before immunoblot analysis was performed using antibodies against PAL (rabbit polyclonal antibody provided by ABclonal).

### Glutathione S-Transferase Pull-Down Assay

To produce glutathione S-transferase (GST)-KFB20 and GST-KFB39, the full-length coding sequences of *KFB20* and *KFB39* were separately amplified and cloned into the pGEX-4T-1 vector. To produce PAL1-His, the coding sequence of *PAL1* was amplified and cloned into the pET-28a (+) vector. Protein expression was induced in the *Escherichia coli* BL21 (DE3) cell line by 0.3 mM IPTG and expressed GST fusion protein was purified using GST resin (C600031, Sangon Biotech, China) and expressed His fusion protein was purified using Ni-NTA agarose (30210, Qiagen, Germany).

To test the influence of CA on the interaction between PAL and KFB proteins, the GST pull-down assay was performed, and 140 μg GST-KFB20 or GST-KFB39 with 100 μg PAL1-His were added into the 1 mL reaction buffer (50 mM Tris-HCl, pH 7.5, 150 mM NaCl, 0.5 mM Na_2_EDTA, 10% glycerol, 0.1% Triton X-100, 1 × complete protease inhibitor cocktail, 1 mM DTT) containing different concentrations of CA, respectively. GST resin was used to bind GST-KFB20 or GST-KFB39. Proteins captured by GST resin were detected by immunoblotting with anti-His antibody (D410002, Sangon Biotech, China) and the Ponceau S staining method.

## Results

### Exogenous Coniferyl Alcohol Inhibits the Growth of Arabidopsis Seedlings

To determine the endogenous amount of lignin monomers, we quantified the three free forms of monolignols and their corresponding aldehydes in Arabidopsis. Although the aldehydes were barely detectable, *p*-coumaryl alcohol and CA accumulated to significant levels, approximately 7 nmol/g fresh weight for CA in stem tissue (Table 1). The lower levels of sinapyl alcohol may be due to the formation of other sinapate derivatives such as sinapoylmalate, sinapoylglucose, etc. in Arabidopsis (Fraser and Chapple, 2011).

**TABLE 1:** Monolignol metabolite contents in various Arabidopsis tissues.

	Mean concentration (pmol g^–1^ fresh weight)		
Metabolite	Leaf	Root	Stem
*p*-Coumaraldehyde	0.64 ± 0.06	32.41 ± 0.65	8.81 ± 1.84
*p*-Coumaryl alcohol	662.29 ± 13.62	28460.42 ± 907.87	5702.78 ± 166.44
Coniferaldehyde	302.34 ± 23.00	267.12 ± 3.28	195.596 ± 9.52
Coniferyl alcohol	938.89 ± 83.29	28773.57 ± 1151.53	6974.56 ± 369.49
Sinapaldehyde	3.47 ± 1.03	11.11 ± 4.53	12.64 ± 0.96
Sinapyl alcohol	45.61 ± 19.50	142.25 ± 23.47	1087.56 ± 24.83

*Values shown are mean ± SD from two biological replicates (n = 2).*

To investigate the possibility of the lignin monomers as signal molecules, we first treated the Arabidopsis seedlings with coniferyl alcohol (CA) to explore whether the exogenous monolignol affected plant growth. Five-day-old Arabidopsis seedlings were transplanted to 1/2 MS solid medium containing different concentrations of CA, and allowed to grow for 10 days before observing the phenotype. Compared to the mock treatment, seedlings grown on the medium containing CA exhibited reduced growth phenotypes at CA concentrations as low as 0.1 mM (Supplementary Figure 1), within approximately an order of magnitude of the endogenous level of CA in Arabidopsis (Table 1).

### Exogenous Coniferyl Alcohol Specifically Affects the Expression of Lignin Biosynthesis Genes

**FIGURE 1:**
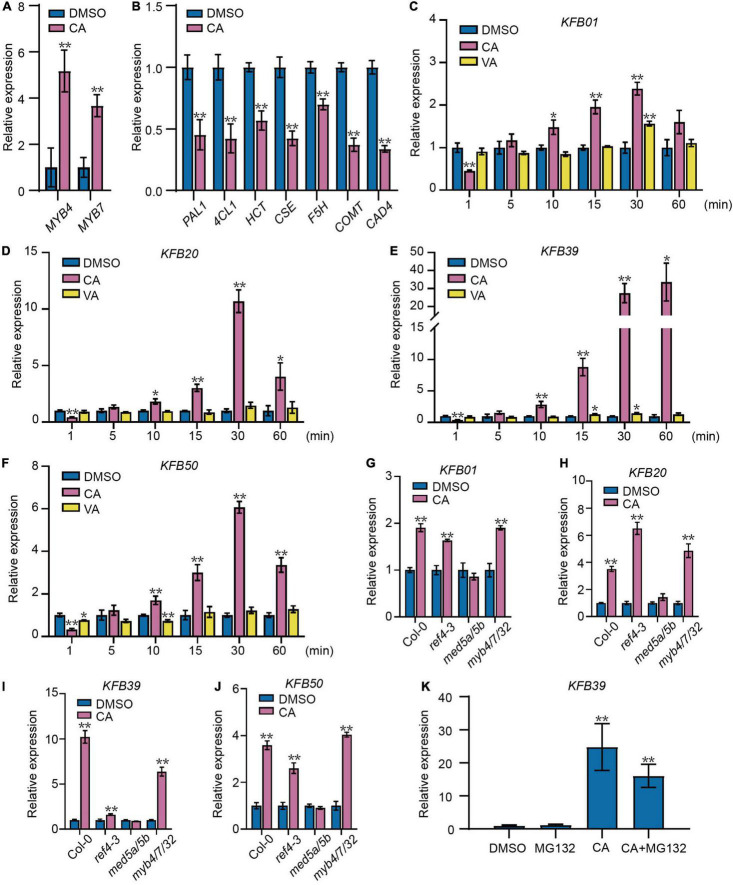
Coniferyl alcohol acts as a signal molecule to regulate the phenylpropanoid pathway. **(A)** The expression of *MYB4/7* was induced by coniferyl alcohol (CA). The transcript levels of *MYB4/7* were assayed in 2-week-old Col-0 seedlings treated with solvent DMSO or 0.1 mM CA for 15 min. **(B)** Relative transcript levels of lignin biosynthetic genes are decreased after CA treatment. The transcript levels of *PAL1*, *4CL1*, *HCT*, *CSE*, *F5H*, *COMT*, and *CAD4* were assayed in 2-week-old Col-0 seedlings treated with solvent DMSO or 0.1 mM CA for 4 h. qRT-PCR assays of *KFB01* **(C)**, *KFB20* **(D)**, *KFB39* **(E),** and *KFB50* **(F)** in Col-0 seedlings. **(C–F)** Two-week-old Col-0 seedlings were separately treated with solvent DMSO, 0.1 mM VA (vanillyl alcohol) or 0.1 mM CA for different time intervals. qRT-PCR assays showing the expression of *KFB01* **(G)**, *KFB20* **(H)**, *KFB39* **(I),** and *KFB50* **(J)** in response to CA in Col-0, *ref4-3, med5a/5b* and *myb4/7/32* seedlings. **(G–J)** Two-week-old seedlings were treated with solvent DMSO or 0.1 mM CA for 15 min. **(K)** The induction of *KFB39* expression by CA does not rely on proteolysis. The transcript level of *KFB39* after 1 h treatment with 0.1 mM CA with or without 60 μM MG132 in 2-week-old Col-0 seedlings. All data represent average transcript level values (±SD) relative to DMSO-treated plants. Asterisks indicates a statistically significant difference between the treated and DMSO control groups (Student’s *t*-test, *P < 0.05, **P < 0.01, *n* = 3).

To explore the mechanism of CA inhibition of the growth of Arabidopsis seedlings, we performed RNA sequencing (RNA-seq) analysis on the 14-day-old Col-0 seedlings after different times of 0.1 mM CA treatment, and differentially expressed genes with FDR < 0.001 and fold-change ≥ 2 or ≤ 0.5 were identified. Compared with the ethanol control, 1124, 1243, 1188, and 1353 genes were up-regulated respectively, while 1139, 1195, 1122, and 3269 genes were down-regulated after 15, 30 min, 1, and 4 h treatment of CA respectively (Supplementary Data Sets 1–4). CA could specifically induce the expression of lignin biosynthesis transcriptional repressors *MYB4* and *MYB7* after only 15 min treatment, while the transcript levels of the lignin biosynthesis genes *PAL1*, *4-coumarate:CoA ligase 1* (*4CL1*), *hydroxycinnamoyl transferase* (*HCT*), *caffeoyl shikimate esterase* (*CSE*), *ferulate 5-hydroxylase* (*F5H*), *caffeic acid 3-O-methyltransferase* (*COMT*) and *cinnamyl alcohol dehydrogenase* (*CAD4*) were all significantly decreased at 4 h after exposure to CA (Figures 1A,B). The results suggested that CA treatment caused the down-regulation of lignin biosynthesis genes by inducing the expression of *MYB4/7*.

### The Expression of *KFB01/20/39/50* Rapidly Responds to Coniferyl Alcohol

Interestingly, genes encoding the Kelch repeat F-box (KFB) proteins KFB01/20/39/50 that regulate the protein stability of PAL (Zhang et al., 2013, 2015) were also rapidly induced by CA treatment (Figures 1C–F). The transcript levels of *KFB39* was significantly induced after as soon as 10 min, and induced by 30-fold after 30 min treatment. The expression of other *KFB* genes was induced to a lesser extent (Figure 1C). To determine the specificity of CA induction of the *KFB* genes, seedlings were also treated with vanillyl alcohol (VA), which has a similar chemical structure to CA but with a shortened side-chain (Supplementary Figure 2). qRT-PCR analyses showed that the transcripts of *KFB* genes were induced as early as 10 min by CA but not VA treatment or solvent control (Figures 1C–F). We also compared the ability of other monolignols (*p*-coumaryl alcohol, caffeyl alcohol, and sinapyl alcohol) to induce *KFB39* transcripts. *p*-Coumaryl alcohol and sinapyl alcohol significantly induced *KFB39* transcripts, but caffeyl alcohol, the precursor of C-lignin only reported to date in some seed coats (Chen et al., 2012), did not (Supplementary Figure 3). This result suggests that all the classical monolignols found in vegetative tissues are signaling molecules of lignin biosynthesis through induction of the *KFB* genes.

To test if the induction of the *KFB* genes by CA treatment is dependent on MYB4 and/or its homologs MYB7 and MYB32, the triple mutant *myb4/7/32* was treated with CA or dimethyl sulfoxide (DMSO) as control. *KFB* gene transcript levels were enhanced in *myb4/7/32* by CA treatment (Figures 1G–J), indicating that CA induction of the *KFB* genes is independent of MYB4 and its homologs.

The Mediator complex subunits MED5a and MED5b have been shown to be involved in the regulation of lignin biosynthesis and also in the expression of the *KFB* genes (Bonawitz et al., 2014; Kim et al., 2020; Wang et al., 2020). The transcript levels of *KFB39* and *KFB50* were significantly decreased in the *med5* mutant. To determine whether MED5 is involved in monolignol signaling, the *med5a/5b* and *ref4-3* (a semidominant point mutation in *MED5a*) were treated with CA. *KFB* transcripts were not induced in *med5a/5b* and *KFB39* transcripts were increased only weakly in *ref4-3* (Figures 1G–J). Therefore, we conclude that MED5a and MED5b are essential for the expression of *KFB* genes, even though they may not be required for CA sensing.

### The Induction of *KFB39* Expression by Coniferyl Alcohol Does Not Require Proteolysis

26S Proteasome-mediated proteolysis is a predominant route for protein degradation (Vierstra, 2009). To test whether the induction of *KFB39* expression by CA is dependent on proteolysis, we performed qRT-PCR analysis on 2-week-old Col-0 seedlings after 1 h treatment with 0.1 mM CA with or without 60 μM MG132 (26S proteasome inhibitor). The results showed that the expression of *KFB39* was significantly induced by CA, while the addition of MG132 had no significant effect on the induction (Figure 1K), indicating that the induction of *KFB39* expression by CA does not require proteolysis.

### The Induction of *KFB39* Expression by Coniferyl Alcohol May Not Rely on Protein Phosphorylation or Dephosphorylation

**FIGURE 2:**
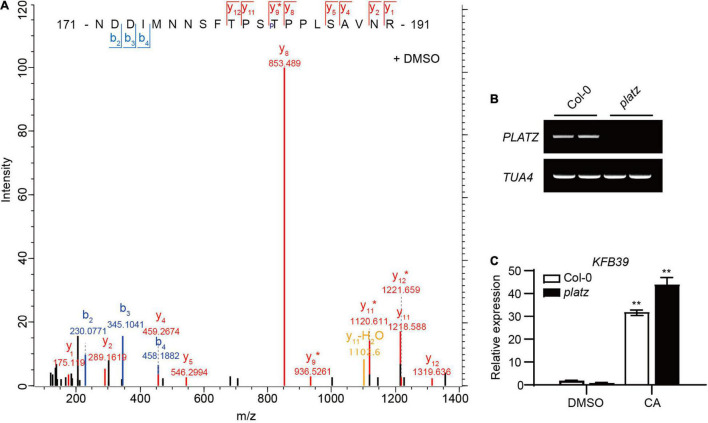
Coniferyl alcohol can induce the up-regulation of *KFB39* expression in *platz*. **(A)** Mass spectrometric analysis of PLATZ phosphorylation *in vivo* at Thr^183^ in 2-week-old Col-0 seedlings treated with solvent DMSO for 1 h. **(B)** RT-PCR result showing that the *platz* was a knock-out mutant line for *PLATZ*. *TUA4* was used as the positive control. **(C)** The expression level of *KFB39* in 14-day-old Col-0 and *platz* seedlings treated with solvent DMSO or 0.1 mM CA for 30 min. All of the data represent average transcript level values (± SD) relative to corresponding DMSO-treated lines. Asterisks indicate a statistically significant difference between the treated and DMSO control groups (Student’s *t*-test, *P < 0.05, **P < 0.01, *n* = 3).

The above studies showed that the expression of *KFB39* can be rapidly induced by CA, the mechanism of which is still unclear. Protein phosphorylation is involved in many signal transduction processes by brassinosteroids, cytokinins, etc. (Hwang et al., 2012; Manghwar et al., 2022). In order to determine whether the induction of *KFB39* by CA relied on phosphorylation modification of some transcription factors, we conducted a phosphorylation proteome study on the nuclear-localized proteins from the 2-week-old Col-0 seedlings treated with solvent DMSO or 0.1 mM CA for 1 h. The results showed that the phosphorylation of the threonine residue at position 183 of the transcription factor PLATZ (AT1G32700) disappeared after the seedlings were treated with CA for 1 h, and dephosphorylated PLATZ cannot be enriched by binding to TiO_2_ beads, while the phosphorylated PLATZ from DMSO treated seedlings can be enriched in this way (Figure 2A). In addition, data on the website ATTED-II v11^1^ show that *PLATZ* is co-expressed with *KFB01*, *KFB20*, *KFB39* and *KFB50* (Obayashi et al., 2022). In order to investigate whether *PLATZ* is involved in CA-mediated *KFB39* induction we ordered a mutant line for *PLATZ* (Figure 2B), and examined the expression level of *KFB39* in the 2-week-old Col-0 and *platz* seedlings after 30 min of solvent DMSO or 0.1 mM CA treatment. The results showed that the induction of *KFB39* expression by CA was not significantly different between Col-0 and *platz* (Figure 2C). Thus we conclude that the induction of *KFB39* expression by CA does not rely on changes in the protein phosphorylation status of PLATZ. However, this does not rule out the possibility that phosphorylation is involved in the CA signal transduction, a possibility that could be studies by future quantitative proteomic studies of CA-responsive proteins.

### L-Phenylalanine Ammonia-Lyase Interact With KFB01/20/39/50

It has been previously reported that KFB01/20/39/50 physically interact with PALs (Zhang et al., 2013, 2015). In order to fully confirm the protein-protein interaction between PAL1/2/3/4 and KFB01/20/39/50, we conducted Co-IP assays using *N. benthamiana* leaves to transiently express the proteins. The full-length KFBs-YFP and PALs-HA were transiently expressed, and we found that PAL1 and PAL4 can interact with the four KFBs (Supplementary Figures 4A,D). Owing to the weak interaction between PAL2 and PAL3 with the KFBs, we constructed the truncated KFBs (KFBs-ΔF) without the F-box motif in KFBs, and found that PAL2 and PAL3 can interact with the four truncated KFBs (Supplementary Figures 4B,C). Due to the larger than expected molecular weight of KFB50-YFP in the Western blot, mass spectrometry analysis was performed. The results showed that the band was indeed KFB50-YFP, and the covalent modification by the tobacco protein polyubiquitin 11-like (Gene ID: 107766892) caused the larger molecular weight than expected (Supplementary Table 2). It can therefore be concluded that the PAL1/2/3/4 interact with the four KFBs.

### Exogenous Coniferyl Alcohol Affects the Stability of L-Phenylalanine Ammonia-Lyase Mainly Through KFB39 and KFB50

It has been reported that KFB01, KFB20, KFB39, and KFB50 redundantly control PAL stability (Zhang et al., 2015). To generate the quadruple mutant, the previously published *kfb01/20/50* triple mutant and the T-DNA mutant line for *KFB39* were crossed, and RT-PCR analysis confirmed that the expression of all four *KFBs* was disrupted in *kfb01/20/39/50* (Supplementary Figure 5).

**FIGURE 3:**
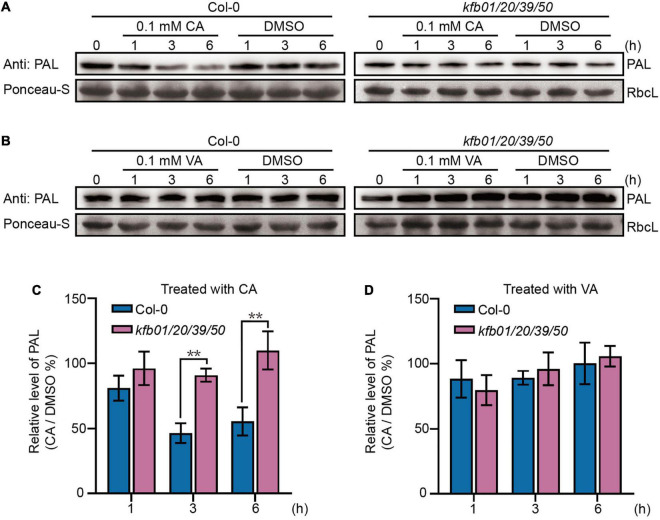
Exogenous CA can specifically induce the degradation of PAL proteins in Arabidopsis. **(A)** Exogenous CA can induce the degradation of PAL proteins in Col-0 but not *kfb01/20/39/50*. **(B)** Exogenous VA has no effect on the stability of PAL proteins in Col-0 and *kfb01/20/39/50*. For **(A,B)**, 2-week-old seedlings were separately treated with solvent DMSO, 0.1 mM CA or 0.1 mM VA in 1/2 MS liquid medium for 1, 3, and 6 h, and the solvent DMSO was used as control. Ponceau staining of RbcL was shown as loading control. **(C,D)** Quantitative analysis of the band intensity in **(A,B)**. Protein contents were normalized to the corresponding content of RbcL by Ponceau staining. The relative level of PAL (CA/DMSO%) at 0 min was set to 100%. Error bars are means ± SD (Student’s *t*-test, **P < 0.01, *n* = 3).

Because of the rapid up-regulation of *KFBs* by exogenous CA, we hypothesized that exogenous CA may trigger PAL degradation. To test this possibility, an anti-PAL antibody was developed against the two peptide sequences that are conserved among the four PAL proteins in Arabidopsis (Supplementary Figure 6A), and the 2-week-old Col-0, *pal2/3/4*, *PAL3-GFP/Col-0* and *kfb01/20/39/50* seedlings were used to detect and prove the specificity of the antibody (Supplementary Figure 6B). Then the level of immuno-detectable PAL protein was compared in extracts of 2-week-old Col-0 and *kfb01/20/39/50* seedlings after 1, 3, and 6 h of CA, VA or solvent DMSO treatment. The results showed that the protein level of PAL in Col-0 seedlings decreased after 3 and 6 h of CA treatment, but remained stable after VA treatment, while the protein level of PAL in *kfb01/20/39/50* seedlings remained stable after CA or VA treatment (Figure 3).

**FIGURE 4:**
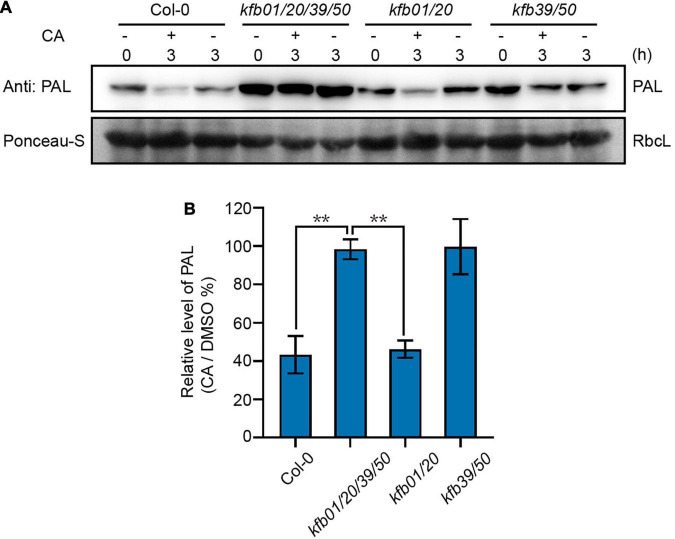
Exogenous CA induces the degradation of PAL proteins mainly through KFB39 and KFB50. **(A)** The protein level of PAL in Col-0, *kfb01/20/39/50*, *kfb01/20*, and *kfb39/50* seedlings after CA treatment. Two-week-old Col-0, *kfb01/20/39/50*, *kfb01/20*, and *kfb39/50* seedlings were treated with 0.1 mM CA for 3 h and the solvent DMSO was used as control. Ponceau staining of RbcL was shown as loading control. **(B)** Quantitative analysis of the band intensity in **(A)**. Protein contents were normalized to the corresponding content of RbcL by Ponceau staining. The relative level of PAL (CA/DMSO%) at 0 min was set to 100%. Error bars are means ± SD (Student’s *t*-test, **P < 0.01, *n* = 3).

Since the expression of *KFB39* was affected by CA induction the most, and *KFB01* to a lesser extent, we generated the *kfb1/20* and *kfb39/50* double mutant based on the homology relationship of the four KFBs (Zhang et al., 2015). Then the protein level of PAL was determined in the 2-week-old Col-0, *kfb01/20/39/50*, *kfb01/20* and *kfb39/50* seedlings after 3 h of solvent DMSO or 0.1 mM CA treatment. The results showed that the protein level of PAL in the Col-0 and *kfb01/20* seedlings decreased to approximately 40%, but remained unchanged in *kfb01/20/39/50* and *kfb39/50* seedlings after 3 h of CA treatment (Figure 4). Therefore, CA-induced PAL degradation mainly depends on KFB39 and KFB50.

### Coniferyl Alcohol Has No Effect on the Interaction Between Kelch Repeat F-box and L-Phenylalanine Ammonia-Lyase

**FIGURE 5:**
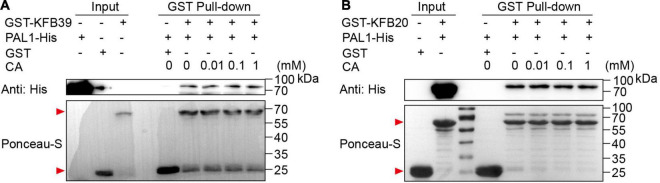
Coniferyl alcohol has no effect on the interaction between KFBs and PAL1 in GST pull-down assays. Lack of effect of CA on the physical interaction of PAL1-His with GST-KFB39 **(A)** or GST-KFB20 **(B)** *in vitro* was verified by GST pull-down assays. PAL1-His and GST-KFB39 **(A)** or GST-KFB20 **(B)** were incubated in binding buffer containing different concentrations of CA, and GST resin was washed for five times and eluted. His-tagged protein was immunoblotted using anti-His antibody and GST-tagged protein was stained by Ponceau S. Red arrows indicate GST-tagged proteins.

It has been reported that ligands can promote the interaction between F-box receptors and their substrates, such as the receptor of auxin TIR1 (Dharmasiri et al., 2005; Kepinski and Leyser, 2005). In order to investigate whether the affinity between KFBs and PAL proteins is affected in the presence of the small molecule CA, we carried out *in vitro* GST pull-down assays. PAL1-His, GST-KFB20 and GST-KFB39 were separately purified from *E. coli* host, and GST pull-down assays with different concentrations of CA were conducted. The results showed that the affinity between KFB20 and KFB39 with PAL1 remained unchanged under the treatment of different concentrations of CA (Figure 5), indicating that CA has no effect on the interaction between KFBs and PAL proteins.

### Mutants With Compromised Coniferyl Alcohol Biosynthesis Have Higher L-Phenylalanine Ammonia-Lyase Accumulation

**FIGURE 6:**
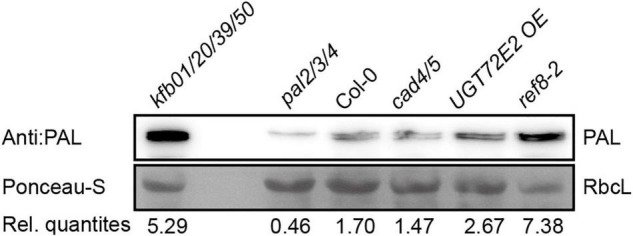
The protein level of PAL is increased in *ref8-2* and *UGT72E2 OE* seedlings. The protein level of endogenous PAL in 2-week-old Col-0, *cad4/5*, *UGT72E2 OE* and *ref8-2* seedlings. Endogenous PAL content was normalized to the content of RbcL by Ponceau S staining, and relative quantities (Rel. quantities) represent the ratio of the intensity of PAL relative to the corresponding RbcL band.

Based on the above-mentioned experimental results, we speculated that the content of CA in plants has impact on the protein level of endogenous PAL. Cinnamyl alcohol dehydrogenases (CADs) are required for the reduction of aldehydes to monolignols, and the disruption of *CAD4/5* results in weakly reduced CA in Arabidopsis (Jourdes et al., 2007; Anderson et al., 2015). *UGT72E2* encodes a glycosyltransferase which catalyzes the formation of monolignol glucosides from monolignols, and the CA content in *UGT72E2 OE* is slightly lower than that in Col-0 (Lanot et al., 2006; Konig et al., 2014). *C3’H* encodes *p*-coumaroyl shikimate 3’ hydroxylase, and *ref8* with compromised C3’H activity exhibits sharply reduced lignin content compared to Col-0 (Kim et al., 2011; Bonawitz et al., 2014). We examined the relative content of endogenous PAL in 2-week-old Col-0, *cad4/5*, *UGT72E2 OE* and *ref8-2* seedlings. As shown in Figure 6, the protein level is slightly elevated in *UGT72E2 OE*, but at similar level in *cad4/5*. In sharp contrast, PAL protein accumulation is significantly higher in *ref8-2*. These data suggest that plants with reduced CA biosynthesis have higher PAL protein content.

## Discussion

In this study, we have shown that CA, the major monolignol, can function as a signal molecule to regulate the phenylpropanoid pathway in multiple ways. CA treatment can rapidly induce the expression of the genes encoding Kelch repeat F-box proteins, which mediate PAL stability. On the other hand, the expression of the lignin biosynthetic genes was feedback-regulated by CA, and this may be caused by CA-induced *MYB4/7*.

### KFB01/20/39/50 Play Different Roles in Regulating L-Phenylalanine Ammonia-Lyase Protein Stability

It has been previously reported that KFB01, KFB20, KFB39, and KFB50 all interact with PALs and mediate the protein stability. However, protein sequence alignment suggests that KFB01 and KFB20 are closer than the other two (Zhang et al., 2015). The response of *KFB39* expression to CA is much more rapid and striking compared to the other *KFB*s (Figures 1C–F). As shown in Figure 4, KFB39 and KFB50 are the more important in terms of CA-mediated PAL degradation, whereas CA-induced *KFB01/20* contributes little to PAL degradation mediated by CA. This may be due to the fact that the expression levels of these *KFBs* are quite different after CA treatment, especially *KFB39*, although it is not clear why the induction of *KFB01*, *KFB20*, *KFB39* and *KFB50* is different.

### Coniferyl Alcohol-Induced *MYB4/7* Play Important Roles in Regulating the Phenylpropanoid Pathway

MYB4 and its homolog MYB7 play important roles in the regulation of the phenylpropanoid pathway in Arabidopsis, and many other plant species (Jin et al., 2000; Shen et al., 2012; Fornalé et al., 2014). Here we show that the expression of *MYB4/7* can be rapidly and sustainably induced by exogenously applied CA, leading to inhibition of the expression of the lignin biosynthesis genes *PAL1*, *4CL1*, *HCT*, *CSE*, *F5H*, *COMT*, and *CAD4* after 4 h of CA treatment. The response of the lignin biosynthetic genes to CA treatment is much less rapid compared to that of KFB genes, suggesting that the response of the lignin biosynthetic genes is indirect.

### Sensing Coniferyl Alcohol Could Be a Way to Balance Phenylalanine Deamination With Protein Synthesis

L-Phenylalanine ammonia-lyase catalyzes the first step in phenylpropanoid metabolism in which phenylalanine undergoes deamination to yield *trans*-cinnamic acid. Phenylalanine-derived metabolites can constitute up to 30% carbon fixed by photosynthesis. Even though it is well-documented that lignin biosynthesis is subjected to tight regulation, how plants sense monolignol production is not clear. We showed that exogenous CA treatment can enhance the expression of *KFB39* by 30-fold in 30 min (Figure 1). This leads to PAL degradation and theoretically shifts phenylalanine distribution to protein synthesis. Interestingly, *ref8-2* has much more PAL accumulation compared with wild type controls (Figure 6). We have found that exogenous phenylalanine treatment partially rescued the growth defects of the mutant (data not shown). It is possible that the growth defects of *ref8-2* are due to overconsumption of phenylalanine as a consequence of compromised PAL degradation.

## Data Availability Statement

The original contributions presented in the study are included in the article/Supplementary Material, further inquiries can be directed to the corresponding author/s.

## Author Contributions

QZ conceived the study. QZ, MG, FC, and RD designed the experiments. MG, CL, XS, FC, and SW performed the experimental work. QZ, MG, and RD wrote the manuscript. All authors contributed to the article and approved the submitted version.

## Conflict of Interest

The authors declare that the research was conducted in the absence of any commercial or financial relationships that could be construed as a potential conflict of interest.

## Publisher’s Note

All claims expressed in this article are solely those of the authors and do not necessarily represent those of their affiliated organizations, or those of the publisher, the editors and the reviewers. Any product that may be evaluated in this article, or claim that may be made by its manufacturer, is not guaranteed or endorsed by the publisher.

## Footnotes

1
https://atted.jp/

## Supplementary Material

Supplementary Figure 1 Arabidopsis seedlings are sensitive to exogenous CA. 3-Day-old Col-0 seedlings were separately transplanted to 1/2 MS solid medium supplemented with different concentrations of CA or solvent DMSO control for 14 days. Scale bar, 2 cm.

Supplementary Figure 2 Chemical structures of coniferyl alcohol and vanillyl alcohol.

Supplementary Figure 3 Lignin monomers induce *KFB39* expression. Two-week-old Col-0 seedlings were treated with solvent EtOH and 0.1 mM C (caffeyl alcohol), H (*p*-coumaryl alcohol), G (coniferyl alcohol), S (sinapyl alcohol) in 1/2 MS liquid medium for 15 min. Data represent average gene expression (transcript level) values (±SD) relative to EtOH-treated plants. Asterisk indicates a statistically significant difference between treatments (Student’s *t*-test, **P* < 0.05, ^**^*P* < 0.01, *n* = 3).

Supplementary Figures 4 YFP-tagged KFB01/20/39/50 can interact with HA-tagged PAL1/2/3/4 in Co-IP analyses. The Co-IP assay revealed that YFP-tagged full-length KFB01/20/39/50 interact with HA-tagged PAL1 **(A)** and PAL4 **(D)**, and YFP-tagged truncated KFB01/20/39/50 interact with HA-tagged PAL2 **(B)** and PAL3 **(C)**. Owing to the low expression level of KFB20-ΔF-YFP, we use YFP-KFB20-ΔF in this Co-IP experiment. The IP (KFB01/20/39/50) and Co-IP (PAL1/2/3/4) signals were tested by Western blot assays with anti-GFP and anti-HA antibodies, respectively. Specific bands are indicated by red arrows.

Supplementary Figure 5 Schematic diagram of T-DNA insertion sites in *kfb01/20/39/50* quadruple mutant. **(A)** Schematic representation of *kfb01, kfb20, kfb39*, and *kfb50* T-DNA inserted loss-of-function mutant alleles. Inverted triangles depict T-DNA insertion sites. **(B)** RT-PCR analysis of *KFB01, KFB20, KFB39*, and *KFB50* transcripts in the 14-day-old seedlings of Col-0 and *kfb01/20/39/50* quadruple mutant. *TUA4* was used as the positive control.

Supplementary Figure 6 Peptide sequences and the specificity of endogenous PAL antibody. **(A)** Amino acid sequences alignment of PAL1/2/3/4 and the two peptide sequences used to produce PAL antibody. **(B)** Two-week-old seedlings of Col-0, *pal2/3/4*, *PAL3-GFP* and *kfb01/20/39/50* were used for Western blot assay to test the specificity of PAL antibody. Protein bands marked by red triangles were endogenous PAL proteins (lower band) and PAL3-GFP (upper band). Ponceau S staining of RbcL was shown as loading control.

Supplementary Table 1 Primers used in RT-PCR and qRT-PCR analyses.

Supplementary Table 2 Proteins in SDS-PAGE gel blocks identified by mass spectrometry.
