# Supplementary figures and images for: Dual Mechanisms of Coniferyl Alcohol in Phenylpropanoid Pathway Regulation
Source: Front Plant Sci. 2022 May 6;13:896540. doi: 10.3389/fpls.2022.896540 (PMC9121011; doi:10.3389/fpls.2022.896540)

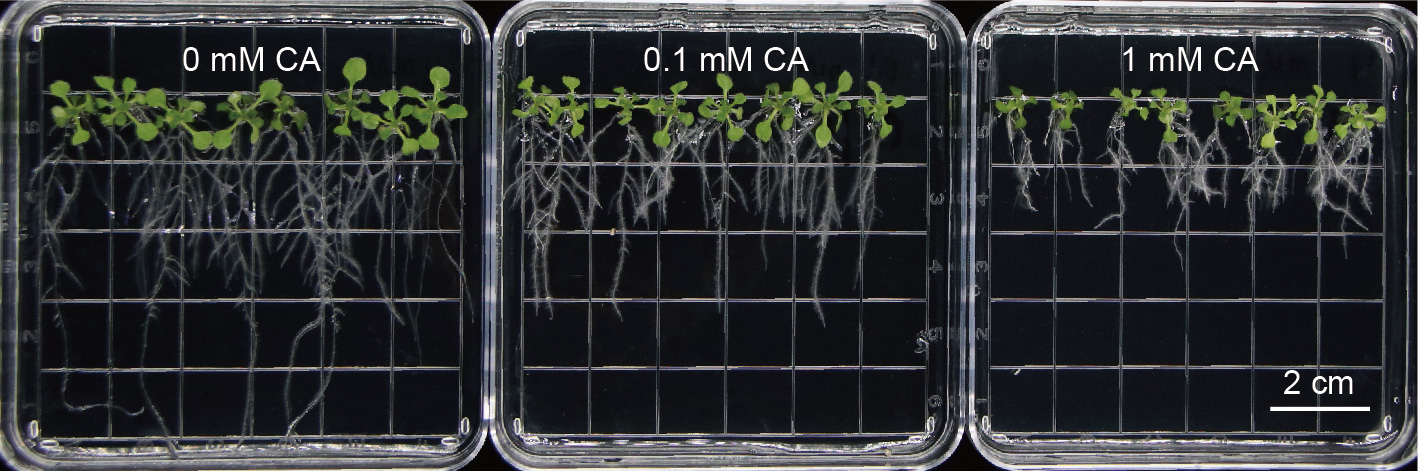

Supplement: Supplementary Figure 1 — Arabidopsis seedlings are sensitive to exogenous CA. 3-Day-old Col-0 seedlings were separately transplanted to 1/2 MS solid medium supplemented with different concentrations of CA or solvent DMSO control for 14 days. Scale bar, 2 cm. [file Image_1.JPEG]

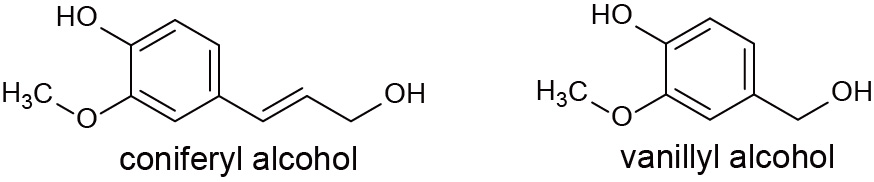

Supplement: Supplementary Figure 2 — Chemical structures of coniferyl alcohol and vanillyl alcohol. [file Image_2.JPEG]

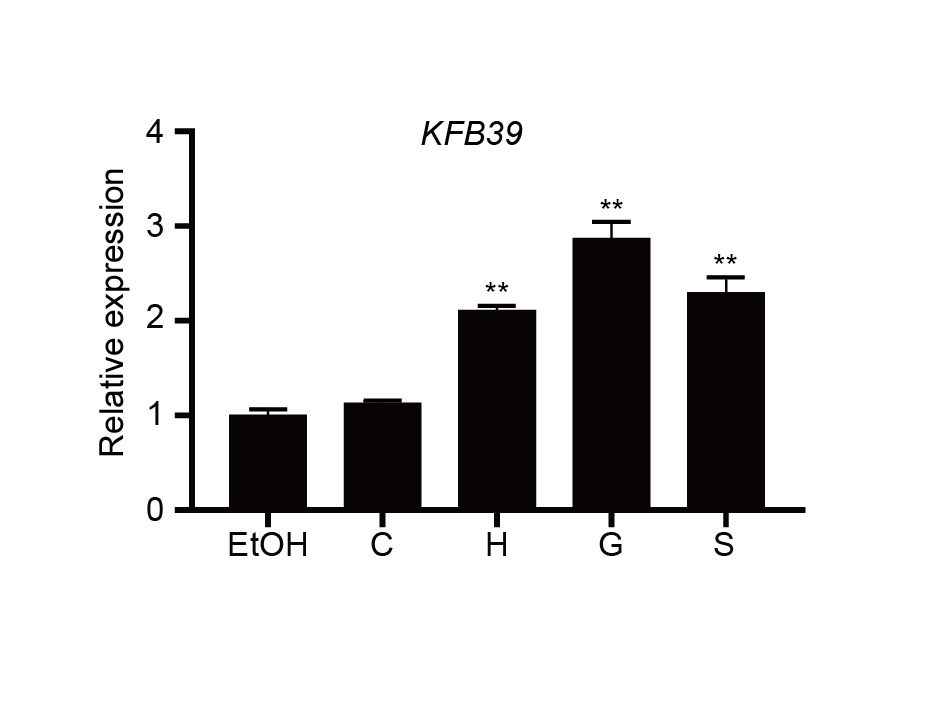

Supplement: Supplementary Figure 3 — Lignin monomers induce KFB39 expression. Two-week-old Col-0 seedlings were treated with solvent EtOH and 0.1 mM C (caffeyl alcohol), H (p-coumaryl alcohol), G (coniferyl alcohol), S (sinapyl alcohol) in 1/2 MS liquid medium for 15 min. Data represent average gene expression (transcript level) values (±SD) relative to EtOH-treated plants. Asterisk indicates a statistically significant difference between treatments (Student’s t-test, *P < 0.05, **P < 0.01, n = 3). [file Image_3.JPEG]

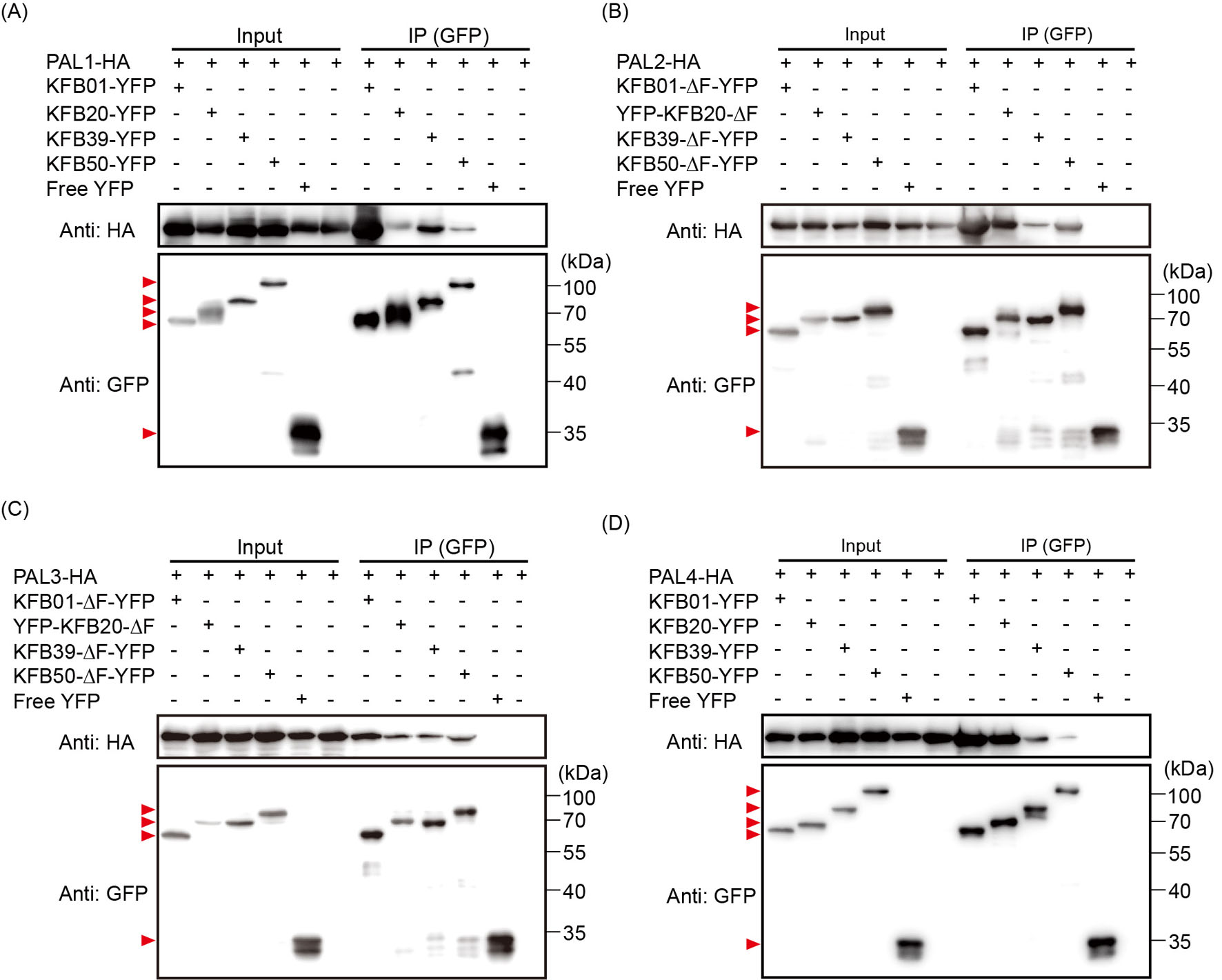

Supplement: Supplementary Figures 4 — YFP-tagged KFB01/20/39/50 can interact with HA-tagged PAL1/2/3/4 in Co-IP analyses. The Co-IP assay revealed that YFP-tagged full-length KFB01/20/39/50 interact with HA-tagged PAL1 (A) and PAL4 (D), and YFP-tagged truncated KFB01/20/39/50 interact with HA-tagged PAL2 (B) and PAL3 (C). Owing to the low expression level of KFB20-ΔF-YFP, we use YFP-KFB20-ΔF in this Co-IP experiment. The IP (KFB01/20/39/50) and Co-IP (PAL1/2/3/4) signals were tested by Western blot assays with anti-GFP and anti-HA antibodies, respectively. Specific bands are indicated by red arrows. [file Image_4.JPEG]

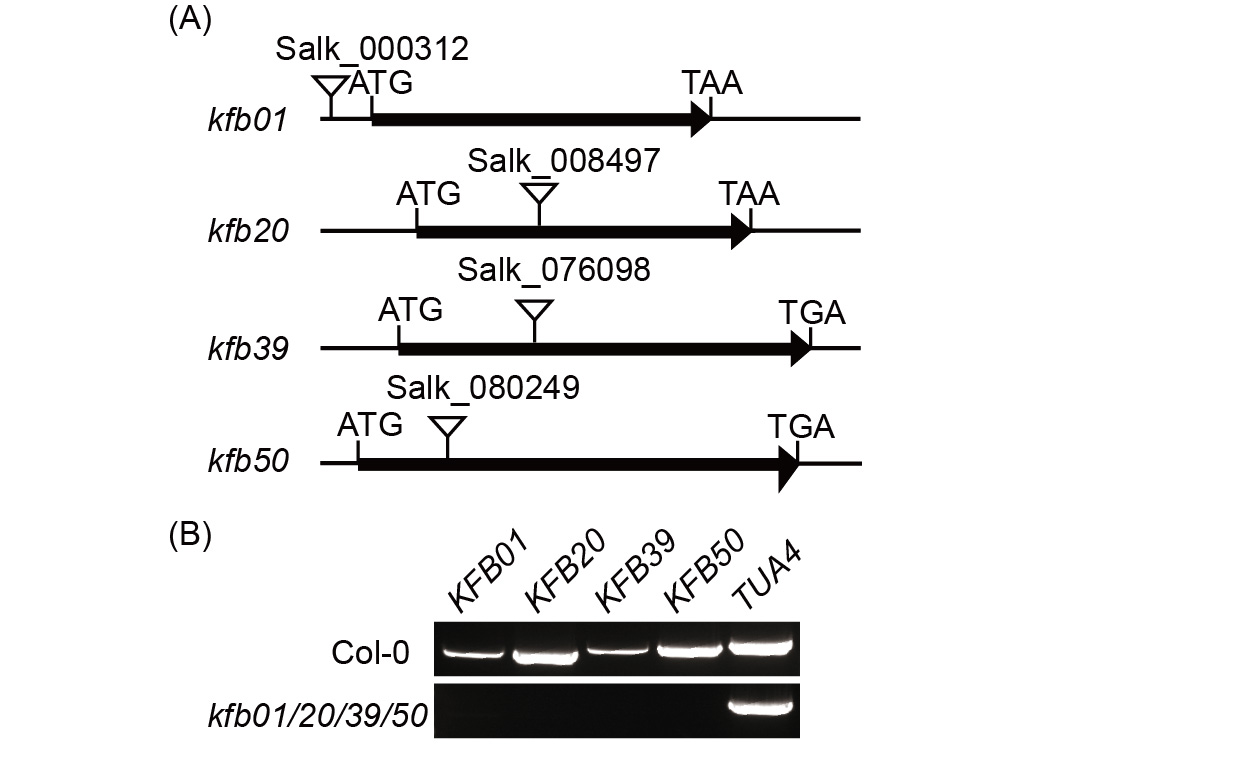

Supplement: Supplementary Figure 5 — Schematic diagram of T-DNA insertion sites in kfb01/20/39/50 quadruple mutant. (A) Schematic representation of kfb01, kfb20, kfb39, and kfb50 T-DNA inserted loss-of-function mutant alleles. Inverted triangles depict T-DNA insertion sites. (B) RT-PCR analysis of KFB01, KFB20, KFB39, and KFB50 transcripts in the 14-day-old seedlings of Col-0 and kfb01/20/39/50 quadruple mutant. TUA4 was used as the positive control. [file Image_5.JPEG]

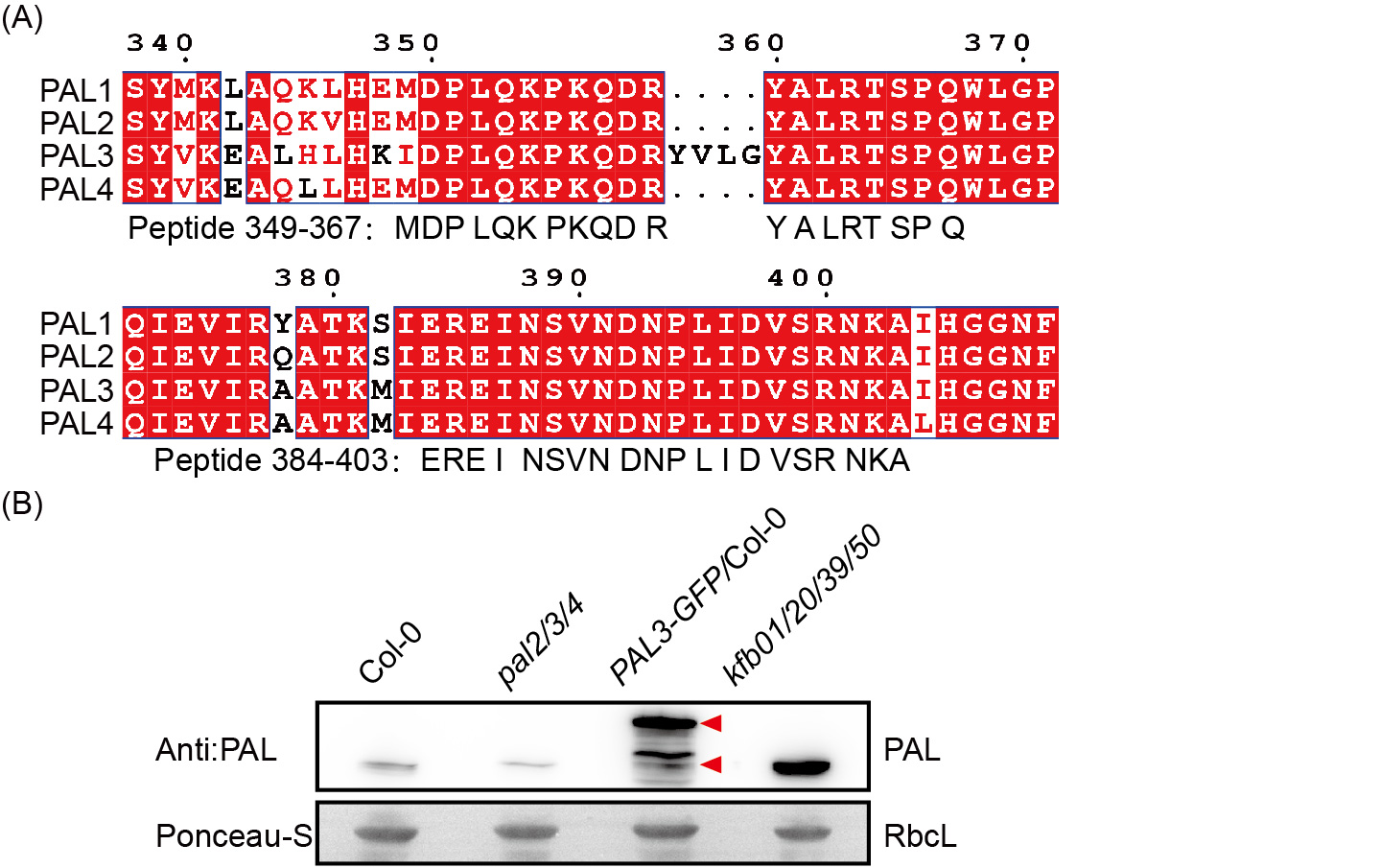

Supplement: Supplementary Figure 6 — Peptide sequences and the specificity of endogenous PAL antibody. (A) Amino acid sequences alignment of PAL1/2/3/4 and the two peptide sequences used to produce PAL antibody. (B) Two-week-old seedlings of Col-0, pal2/3/4, PAL3-GFP and kfb01/20/39/50 were used for Western blot assay to test the specificity of PAL antibody. Protein bands marked by red triangles were endogenous PAL proteins (lower band) and PAL3-GFP (upper band). Ponceau S staining of RbcL was shown as loading control. [file Image_6.JPEG]
