# Supplementary material for: Dual Mechanisms of Coniferyl Alcohol in Phenylpropanoid Pathway Regulation
Source: Front Plant Sci. 2022 May 6;13:896540. doi: 10.3389/fpls.2022.896540 (PMC9121011; doi:10.3389/fpls.2022.896540)
Supplement: Supplementary Table 1 — Primers used in RT-PCR and qRT-PCR analyses. [file Table_1.docx]

**Supplementary Table 1 |** Primers used for gene expression analysis.

| **For RT-PCR identification of mutant** | |
| --- | --- |
| KFB01-F | ATGGAGCTTATTCCTGATCTT |
| KFB01-R | TTATATCTCCAAGAAGCAACC |
| KFB20-F | ATGGAACTTATCCCCAATCTTC |
| KFB20-R | TTAGACCTCCAAGAAGCAGCCA |
| KFB39-F | ATGACTATGGAAGTGTCGAAA |
| KFB39-R | TCAAACATAGATTGAAGCATGCG |
| KFB50-F | ATGGGAGTGTCAAAGAAGAAA |
| KFB50-R | TCAAACGTAGATTGAAGAACACG |
| TUA4-F | ATGAGAGAGTGCATTTCGATC |
| TUA4-R | TTAGTATTCCTCTCCTTCATCAT |
| **For detecting the expression level by qRT-PCR** | |
| qRT-PAL1-F2 | CGCAACGTACCCGTTGATTC |
| qRT-PAL1-R2 | GATCCTGTTCGGGATAGCCG |
| qRT-4CL1-F1 | GGAGTTATCAGAAGATGATGTG |
| qRT-4CL1-R1 | CCATTTGCTAGTTTTGCCCTCA |
| qRT-HCT-F | ATCTGTCAGCCCTTGTCC |
| qRT-HCT-R | ATTCCACCAGGTCCCATA |
| qRT-CSE-F1 | GGGACAGCGGATGGAGTAAC |
| qRT-CSE-R1 | TCTCGTCAGGCTCTCCTTG |
| qRT-COMT-F1 | CATGCTCGACCGTATCCTCC |
| qRT-COMT-R1 | ACCAAGCCCGTAAATCCGTT |
| qRT -F5H-F | TCAGTGAGACAGCGGATCTTCA |
| qRT -F5H-R | ACTTCGGCGAGTTCTTGTTGGA |
| qRT-CAD4-F | AGTCGGCGTGGTCGTTGGAT |
| qRT-CAD4-R | TGCTCCACCGCCATTCCTTCT |
| qRT-GAPDH-F | TCTGACCTTGACATTGTTTCCA |
| qRT-GAPDH-R | TCTCCAGTCCTTCATTGATGG |
| qRT -KFB1-F | GGCAGAAGACAGATGGGCTT |
| qRT -KFB1-R | TGTGGTGACGTCGAACGATT |
| qRT -KFB20-F | ACAAGTGGACGTTTTTGCCG |
| qRT -KFB20-R | TGGCATATCCACCGATGACG |
| qRT -KFB39-F | AGGAGCAGATCCCGCTTTTC |
| qRT -KFB39-R | TAAACGTCTCTCGTCGGCTG |
| qRT -KFB50-F | AAGGTTCAGAAGAGGCAGGC |
| qRT -KFB50-R | ATCCGTCGCCATCGAGAATC |
